# Association of 18bp insertion/deletion polymorphism, at −2549 position of VEGF gene, with diabetic nephropathy in type 2 diabetes mellitus patients of North Indian population

**DOI:** 10.1186/s40200-015-0144-3

**Published:** 2015-03-27

**Authors:** Dnyanesh Amle, Rashid Mir, Alka Khaneja, Sarita Agarwal, Ravinder Ahlawat, Prakash C Ray, Alpana Saxena

**Affiliations:** Department of Biochemistry, Cancer Genetics Laboratory, Maulana Azad Medical College and associated hospitals, New Delhi, 110002 India; PRINCE FAHD BIN SULTAN RESEARCH CHAIR, Division of Molecular Genetics, Faculty of Applied Medical Sciences, University of Tabuk, Tabuk, 71491 Saudi Arabia; Department of Medicine, Maulana Azad Medical College and associated Hospitals, New Delhi, 110002 India

**Keywords:** Diabetic nephropathy, VEGF, Polymorphism

## Abstract

**Background:**

Vascular endothelial growth factor (VEGF) is a potent multifunctional cytokine which plays a key role in the pathogenesis of diabetic micro-vascular complications. Human VEGF gene is said to be highly polymorphic. Insertion/deletion (I/D) polymorphism of the 18 bp fragment at −2549 position of the promoter region in VEGF gene is said to be of particular interest. The study was aimed to evaluate association of Insertion/deletion (I/D) polymorphism of the 18 bp fragment at −2549 position of the promoter region in VEGF gene, with diabetic nephropathy in type 2 diabetes mellitus.

**Methods:**

This cross sectional study enrolled 40 subjects each of diabetic nephropathy (DN), diabetes mellitus without nephropathy (DM) and normal control subjects. DNA was isolated from peripheral blood leukocytes. Genotyping of the VEGF gene insertion/ deletion (I/D) polymorphism was done by the polymerase chain reaction (PCR) methods. The frequency of VEGF alleles and genotype distribution were compared in diabetic nephropathy, uncomplicated diabetic and control groups.

**Results:**

DD genotype and D allele were found to be significantly associated with DN group (p = 0.009 and 0.02 respectively) in comparison to DM group. Also DD genotype conferred significant risk of diabetic nephropathy in DM group (OR = 4.2) (against combined frequency of ID and II genotype) so does D allele 2.09 (against I allele).

**Conclusion:**

DD genotype and D allele in I/D polymorphism at −2549 position of VEGF gene is associated with increased susceptibility to diabetic nephropathy in north Indian population.

## Background

Diabetic nephropathy (DN) is the leading cause of chronic kidney disease in patients being prescribed renal replacement therapy [1] and is associated with increased cardiovascular mortality [2]. 20%-40% of patients with type 2 diabetes mellitus (DM) ultimately develop nephropathy, but the reason why only some and not all patients with diabetes develop this complication is unknown [3,4]. Previously glycemic control and duration of diabetes were said to be the two principle causes of progression to DN, but variable outcomes in patients with relatively same glycemic status and duration of DM have indicated role of environmental factors and genetic susceptibility to the condition [5]. Hyperglycaemia induced complex interactions between metabolic and hemodynamic changes, along with variable genetic predisposition, sets the stage for kidney injury, as for other diabetic complications [6]. Relatively earlier onset of diabetes as compared to their European counterparts and increasing longevity amongst Asian population has been proposed to be leading to increased rate of complications, as a result of prolonged exposure to hyperglycaemia [7]. Evidence of abnormal angiogenesis has been found in patients with DN in both type 1 and type 2 diabetes mellitus [8-10].

Vascular endothelial growth factor (VEGF), a potent multifunctional cytokine and highly conserved homodimeric glycoprotein, has been proposed to play key role in the pathogenesis of diabetic micro-vascular complications by virtue of its role in angiogenesis and micro-vascular permeability [11-13]. VEGF, also known as vascular permeability factor is one of the most potent inducers of micro vascular permeability [14]. VEGF-A (also called VEGF), most predominant of the VEGF isoforms, have been shown to be significantly elevated in type 2 diabetic patients and the levels are positively correlated with urinary albumin creatinine ratio [15]. Role of VEGF is well established in Diabetic retinopathy and has led to therapeutic application of this knowledge. Thus studies association of VEGF in diabetic nephropathy can be beneficial to this patient population. The genetic variations in the VEGF gene can influence levels of VEGF protein expression.

Several polymorphisms in VEGF gene have been studied in respect to DN and some of them have been associated with altered serum and urine levels of VEGF [16-20]. Human VEGF gene spans 16,272 bp on chromosome 6 (6p12). It consists of eight exons and it is said to be highly polymorphic [21]. Insertion/deletion (I/D) polymorphism of the 18 bp fragment at −2549 position of the promoter region in VEGF gene has been implicated in a number of diseases with angiogenic basis and hence is of particular interest [22-24]. Asian Indian is ethnically distinct population with leading number of diabetic patients in the world. Concept of both the “thrifty genotypes” and “thrift phenotype” holds true for Indian population. This has been proposed to cause increased predisposition to Diabetes mellitus and thus to associated complications [25,26]. In this light there is need to study various Genetic associations of diabetes and diabetic complications in Indian population. The study was to evaluate association of Insertion/deletion (I/D) polymorphism of the 18 bp fragment, at −2549 position of the promoter region in VEGF gene, with diabetic nephropathy in north Indian population.

## Methods

### Design and subject

This case control study enrolled 40 subjects each with diabetic nephropathy (DN), type 2 diabetes mellitus without nephropathy (DM) and 40 normal control subjects. Type 2 diabetes was diagnosed on the basis of the WHO criteria [2]. Diabetic nephropathy status was determined on the basis of questionnaires, clinical features and laboratory data. Albumin creatinine ratio was used to diagnose diabetic nephropathy and those having macroalbuminuria (ACR > 300 mg/g of creatinine) were included as diabetic nephropathy subjects. Subjects with history suggestive of urinary tract infection, nephrolithiasis, acute inflammatory diseases, hematologic diseases, neoplastic diseases and pregnancy were excluded from the study. Control subjects were 40 age and sex matched healthy volunteers with no history of diabetes or any major clinical disorders and had normal fasting plasma glucose level. Study was approved by ethical committee Maulana Azad Medical College and associated hospitals, New Delhi-110002, INDIA.

### Methods

After obtaining informed written consent, medical history was elicited. 6 ml of fasting venous blood was obtained after overnight fasting for plasma glucose level, serum urea, creatinine, lipid profile and serum electrolyte estimation. Serum and whole blood (in EDTA containing vial) was stored at −80 degree for PCR. 10 ml of early morning spot urine sample was obtained for urinary microalbumin estimation. Plasma glucose, serum urea, creatinine, HDL, LDL, Triglycerides, Cholesterol level were estimated on Syncron DXC® clinical chemistry analyzer (Beckman Coulter Ltd. USA) by standard methods. Urinary microalbumin was assayed by turbidimetric method and urinary creatinine by Modified Jaffe’s method on Syncron CX5 clinical chemistry analyzer (Beckman Coulter Ltd. USA).

### Determination of the VEGF genotype

The official gene symbol for the gene is VEGF-A, approved name is Vascular endothelial growth factor and location is chromosome6 (6p12). Polymorphism selected for the present study was Insertion/Deletion (I/D) polymorphism of 18 bp fragment at −2549 position of the promoter region in VEGF gene. It was located at the promoter region of VEGF gene based on its functional nature.

High molecular weight genomic DNA was isolated from peripheral blood leukocytes by ‘GENEAID® DNA extraction kit’ (GeneAid Taiwan, Cat. # GEB100). The I/D polymorphism was analyzed using the

- Primers: forward: 5′-GCTGAGAGTGGGGCTGACTAGGTA-3′ and
- Reverse: 5′-GTTTCTGACCTGGCTATTTCCAGG-3′

**Figure 1:**
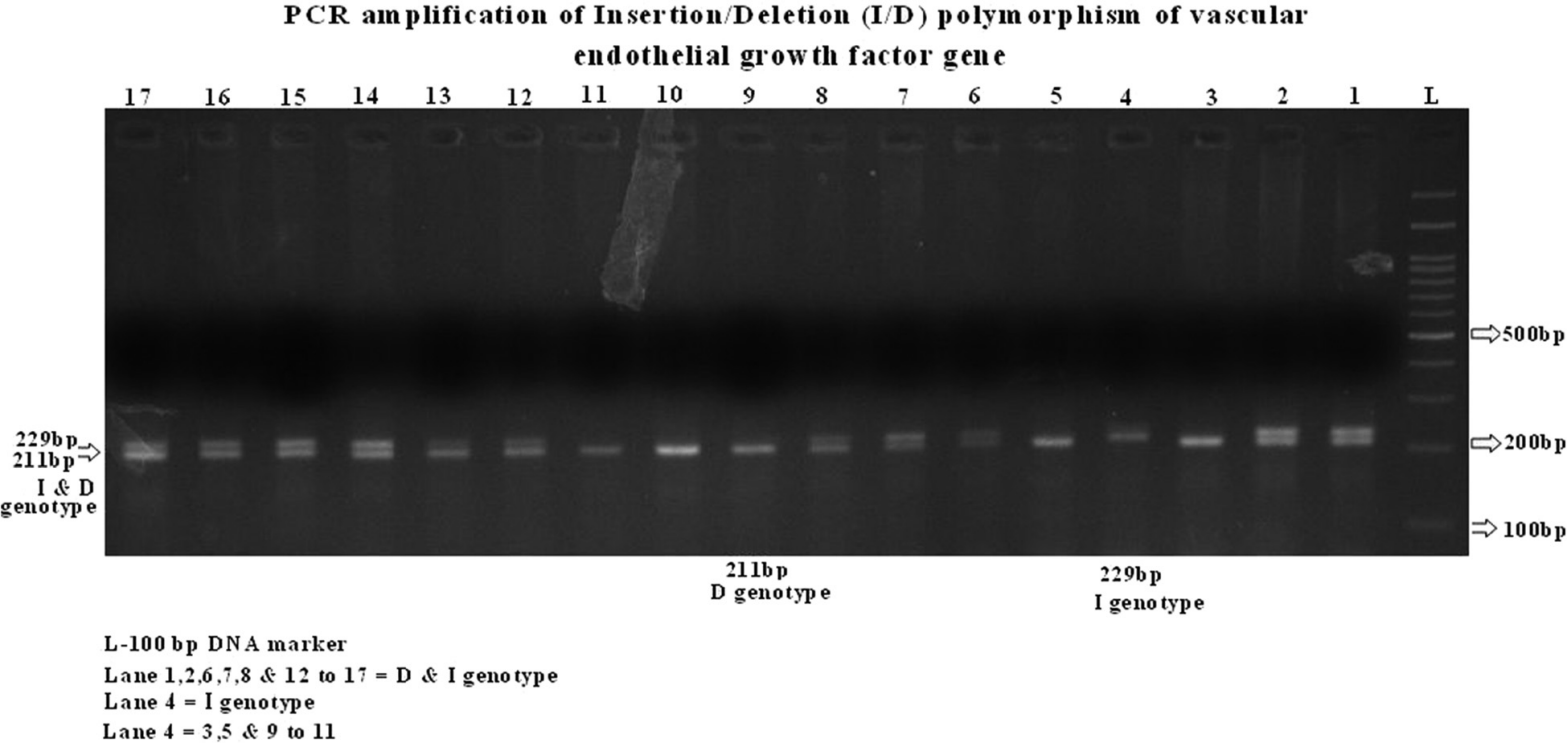
**VEGF Insertion/Deletion (I/D) polymorphism of 18 bp fragment at –2549 position of the promoter region in VEGF gene. Legend**. Lane 1, 2, 6, 7, 8, 12-17 - 211bp and 229bp (I/D). Lane 1, 2, 6, 7, 8, 12-17- 211bp for D. L-100bl DNA ladder.

The amplification was accomplished with a 50 ul reaction mixture containing 5 ul of 20 ng template DNA, 0.25 ul of 25pmol each Primers, 2.5 ul 10 mM dNTPs, 1.5 ul of 20 mM MgCl_2_, 0.3 ul of 5 U/ul Taq polymerase with 2.5 ul of 10X Taq Buffer . The amplification conditions were 6 min of initial denaturation at 95°C; 35 cycles at 94°C for 1min, annealing temperature at 58.8°C for 2min and 72°C for 2min with a final 10 min extension step at 72°C.The amplification products were separated by electrophoresis on 2.5% agarose gel stained with ethidium bromide. For the VEGF I/D polymorphism two bands were observed, 211 bp for D allele and 229 bp for I allele as shown Figure 1

### Statistical analysis

The data were analyzed using the statistical package SPSS (version 15). Quantitative variables were expressed as mean ± standard deviation or median depending on whether the data was normally distributed. Qualitative variables were expressed as number and percentage. Statistical differences between categorical data like gender, genotype distribution were tested using Chi Square test. Independent sample *t* test was used for qualitative variables and normally distributed variables like age, FBS, total cholesterol and triglycerides were analyzed by ANOVA (analysis of variance). Associations of genotypes and alleles were assessed as OR and 95% confidence intervals (CI). Differences by univariate methods (*χ*2 test, unpaired Student *t* test) were analyzed together in a logistic regression analysis to test for significant risk factors for diabetic nephropathy. Values less than or equal to 0.05 were considered statistically significant.

## Results

The distribution of the VEGF DD genotype was significantly different in patients with diabetic retinopathy compared with healthy controls, entire diabetic group and patients with no complications.

Table 1 shows the baseline characteristics of the study population. All the three groups are age (p = 0.78) and sex (p = 0.06) matched. Also there was no statistically significant difference between DM and DN subjects regarding duration of diabetes (0.78). Significant increase in fasting blood sugar (p < 0.0001), total cholesterol (p = 0.46), triglycerides (p < 0.0001), serum urea (p < 0.0001), serum creatinine (p < 0.0001) and urinary albumin creatinine ratio (p < 0.0001) was noted in patients compared to controls.

**Table 1:** **shows the baseline characteristics of the study population**

		**Control (40)**	**Diabetes Mellitus (DM) (40)**	**Diabetic Nephropathy (DN) (40)**	**P value**
Gender	Male	16	18	20	0.06
	Female	24	22	20	
Duration of diabetes			11.2 (8-15)	12.4(10-16)	0.78
Age		50±4.41	50.5±5.5	51±4.4	0.88
FBS (mg %)		84±7.6	166.2±62.2	169±60.2	<0.0001
Total Cholesterol (mg %)		168±25.7	178±33	179±39	0.46
Triglyserides (mg %)		105±28.4	139±48.9	175±81	<0.0001
S. Urea (mg %)		22±5.2	50±5.5	51±4.4	<0.0001
S. Creatinine (mg %)		0.5±0.18	0.71±0.18	1.07±0.8	0.0001
Urinary ACR (mg/g of creatinine)		2.2(0-11)	5.83(0.04-235)	400(310-1.70)	<0.0001

Table 2 showed that there was statistically significant difference in genotype distribution (p = 0.02) and allele frequencies (p = 0.02) of the I/D polymorphism between the three groups in general. Further there was statistically significant difference in DM and DN in terms of both genotypes (0.009) and allels (0.02) with increased frequency of DD genotype (55% Vs 22.5%) and D allele (68.75% Vs 50%) in DN group. Also against controls, DD genotype (p = 0.02, 55%Vs27.5%) and D allele (p = 0.01, 68% Vs 51.25%) frequency were found to be significantly increased in DN group. But such significance of difference was lacking between controls and DM both in terms of genotype and Allelic frequency (p = 0.79 and 0.5 respectively). Odds ratio (OR) was calculated between DN and DM group for risk conferred by DD genotype when compared combined frequency of II and ID genotype (to represent total non DD population). Odds of developing diabetic nephropathy were found to be 4.2 (95% confidence interval 1.28-4.63) in diabetic patients with DD genotype when compared to diabetic population with II and ID genotype together. Odds of developing diabetic nephropathy were found to be 2.2 (95% C.I. = 1.15-4.19) in diabetic patients with D allele when compared to diabetic population with I allele.

**Table 2:** **Genotype and Allele frequencies of VEGF variants in controls , DM and DN groups**

**Genotypes**					**OR (95% C.I.) for DD genotype**
	**II**	**ID**	**DD**	**P value**	
Controls (40)	10 (25)	19 (47.5)	11 (27.5)	0.02	
DM (40)	9 (22.5)	22 (55)	9 (22.5)*		4.2 (1.28-4.63)^a^
DN (40)	7 (17.5)	11 (27.5)	22 (55)**		
**Alleles**					**O.R. (95% C.I.) for for D allele**
	**I**	**D**		**P value**	
Controls (40)	39 (48.75)	41 (51.25)		0.02	
DM (40)	40 (50)	40 (50)^@^			2.2 (1.15-4.19)^b^
DN (40)	25 (31.25)	55 (68.75)^#^			

*p value 0.79 (statistically insignificant) *Vs* controls.

**p value 0.02 and 0.009 *vs* controls and DM group respectively.

^a^DN group *Vs* DM group against combined frequency of II and ID genotype.

^@^p value 0.5 (statistically insignificant) *Vs* controls.

^#^p value 0.01 and 0.02 *vs* controls and DM group respectively.

^b^DN group *Vs* DM group against frequency of I allele.

The power of the present study for detecting the allelic differences between diabetic nephropathy group subjects and controls was calculated. It was found to be significant (96% with α-error = 0.05, two tailed).

## Discussion

In our study we discovered that DD polymorphism of 18 bp fragment at −2549 position of VEGF gene is significantly associated with diabetic nephropathy. We found significantly increased frequency of DD genotype in DN group Vs DM group (p = 0.009), whereas there was no statistically significant increase in frequency of DD genotype in DM group when compared with control group (p = 0.5). Control population here serves to represent the baseline frequency of genotype distribution in normal population, and our finding further emphasizes the fact that those subjects amongst diabetic population had DD genotype were at increased risk of diabetic nephropathy (OR = 4.2, 95% C.I. = 1.28-4.63). The risk was also found to be increased with D allele (OR = 2.21, 95% C.I. = 1.15-4.19) in all subjects with diabetes mellitus (DN + DM). This confers that DD genotype and D allele has increased frequency in DN group than both control and DM group and also is associated with significantly increased risk of diabetic nephropathy.

Explanation to this can be provided by the fact that VEGF by virtue of its property of increasing vascular permeability and angiogenesis plays important role in pathogenesis of diabetic micro-vascular complications. VEGF has been demonstrated in human kidney in podocytes, proximal and distal tubular cells and VEGF receptors are found in pre, post and glomerular region and on renal masengial cells [27]. Hyperglycemia is said to increase VEGF expression by activating protein kinase C (PKC) [28]. VEGF then leads to activation of many pathways and mediates its action by increasing vascular permeability, this particular action along with altered NO synthesis in diabetic milieu can lead to proteinurea [29]. VEGF increases permeability by different mechanism which includes; increasing the diffusion rate of substances across a capillary, widening the junction between endothelial cells and increasing their fenestrations; inducing the formation of vesiculo-vacuolar organelles and caveolae [30]. Other mechanisms proposed are; increased production of α_3_ chain of collagen, plasminogen activators and chemokine attractants, along with recruitment of macrophages [12].

VEGF gene has been found to be associated with diabetic micro vascular complications in studies with VEGF C (−634) G, VEGF (−460), VEGF + 405 and many other polymorphism [11,17,31]. Polymorphisms in promoter region are said to be associated with altered level of serum VEGF [30-34]. Current I/D polymorphism have been to date studied only in two studies, in relation to diabetic nephropathy, to the best of our knowledge [19,20]. Finding of the study by Yang *et al.* [20] goes in favor of our study which says that DD genotype is associated with diabetic nephropathy. Possible explanation given is that D allele is said to be associated with increased transcription of VEGF compared to I allele leading to raised serum levels, further causing the effects. Effect of DD genotype has been also found to be associated with increased serum levels of VEGF by Buraczynska *et al.* [19]. Though the effect was not statistically significant, this was attributed to very small sample size (20 in each group). In contrast to our study Buraczynska et al. [19] reported no significant association of DD genotype in diabetic nephropathy subjects but they have suspected type II error in the study.

## Conclusion

We conclude that, DD genotype and D allele in I/D polymorphism at −2549 position of VEGF gene is associated with increased susceptibility to diabetic nephropathy in north Indian population. Our study is the first study to assess this polymorphism in diabetic nephropathy subjects in Indian population and only second in world to do so to the best of our knowledge. We recommend multicentre studies with larger sample size and including serum VEGF levels to further establish and clarify the role of VEGF and I/D polymorphism in diabetic nephropathy.
